# Enhanced Heat Transfer of 1-Octadecanol Phase-Change Materials Using Carbon Nanotubes

**DOI:** 10.3390/molecules30153075

**Published:** 2025-07-23

**Authors:** Xiuli Wang, Qingmeng Wang, Xiaomin Cheng, Yi Yang, Xiaolan Chen, Qianju Cheng

**Affiliations:** 1 School of Mechatronics and Intelligent Manufacturing, Huanggang Normal University, Huanggang 438000, China; wangxiuli@whut.edu.cn (X.W.); chengxm@whut.edu.cn (X.C.); yangyi@hgnu.edu.cn (Y.Y.); chenxiaolan@hgnu.edu.cn (X.C.); chengqianju@hgnu.edu.cn (Q.C.); 2 School of Materials Science and Engineering, Wuhan University of Technology, Wuhan 430070, China

**Keywords:** 1-Octadecanol, carbon nanotubes, phase-change materials, thermal management

## Abstract

Solid–liquid phase-change materials (PCMs) have attracted considerable attention in heat energy storage due to their appropriate phase-transition temperatures and high thermal storage density. The primary issues that need to be addressed in the wide application of traditional PCMs are easy leakage during solid–liquid phase transitions, low thermal conductivity, and poor energy conversion function. The heat transfer properties of PCMs can be improved by compounding with carbon materials. Carbon nanotubes (CNTs) are widely used in PCMs for heat storage because of their high thermal conductivity, strong electrical conductivity, and high chemical stability. This study investigates the thermal properties of 1-octadecanol (OD) modified with different diameters and amounts of CNTs using the melt blending method and the ultrasonic dispersion method. The aim is to enhance thermal conductivity while minimizing latent heat loss. The physical phase, microstructure, phase-change temperature, phase-transition enthalpy, thermal stability, and thermal conductivity of the OD/CNTs CPCMs were systematically studied using XRD, FTIR, SEM, DSC, and Hot Disk. Moreover, the heat charging and releasing performance of the OD/CNTs CPCMs was investigated through heat charging and releasing experiments, and the relationship among the composition–structure–performance of the CPCMs was established.

## 1. Introduction

Energy storage and thermal management can effectively achieve “peak shaving and valley filling” in terms of time utilization while improving energy efficiency [1,2,3]. Reducing the cost of latent heat storage systems and related materials is a crucial prerequisite for better energy development, making it one of the most promising research fields for lowering energy consumption and a globally recognized focus of study [4]. The principle of latent heat storage primarily relies on the phase-change characteristics of thermal storage media, which absorb or release latent heat during phase transitions to store thermal energy. Materials utilized for latent heat storage are called phase-change materials (PCMs), and the calculation of heat storage capacity of PCMs is shown in Formula (1) [5]. The latent heat storage system with solid–liquid PCMs is considered a very effective method of thermal energy storage. The main drawback of phase-change materials is their low thermal conductivity ranging from 0.2 to 0.8 W/m·K. Therefore, an effective means to enhance the efficiency of heat storage systems is to improve the heat transfer performance of PCMs [6,7].

(1) $$Q=m[C_{sp}(T_{m}-T_{i})+\Delta H+C_{lp}(T_{e}-T_{m})]$$

where Q is the heat storage, m is the mass of the PCMs, and C*_sp_* and C*_lp_* are the specific temperatures of the PCM in solid and liquid forms. ΔH is the phase-transition enthalpy. T_m_ is the melting temperature, and T_i_ and T_e_ are the initial and end temperature.

Latent heat storage technology demonstrates broad research prospects with significant potential for both academic investigation and market applications [8]. This is primarily because phase-change-based thermal storage can achieve high energy storage density within its phase-transition temperature range by utilizing the physical transformation characteristics. However, existing single-component phase-change materials present non-negligible limitations, particularly their low thermal conductivity, which severely restricts energy storage efficiency. This fundamental constraint has become the key bottleneck that must be addressed for the practical implementation of latent heat storage systems. Organic phase-change materials include paraffin, alkanes, esters, fatty acids, alcohols, and various compounds, which have high heat-storage density and low heat-storage cost [9]. The advantage of organic phase-change materials is that they have a low possibility of supercooling during transformation, while their disadvantage is their low thermal conductivity [10,11].

Current research on phase-change thermal storage technology focuses on the development and thermophysical properties of new phase-change materials, where researchers add metal or carbon matrices to PCMs to enhance their thermal conductivity properties [12,13,14]. Carbon materials have high conductivity, low bulk density, good compatibility, and are widely used in practical applications. Their derived structures are very diverse and can be classified by size [15,16,17]. Carbon materials mainly include carbon nanotubes (CNTs), carbon nanowires, carbon nanoparticles, graphene, and other graphene derivatives. Among them, carbon nanotube materials have low density, large specific surface area, and good thermal conductivity, so they have great application prospects [18,19]. Han et al. [20] developed a new phase-change composite based on CNTs loaded with silica aerogels and microencapsulated phase-change materials (MPECMs) through sol–gel and environmental drying. Its enthalpy value is 170 J/g, and the encapsulation efficiency is 72%. At the same time, it maintains the stability of its basic performance after 200 cycles. Wu et al. [21] investigated how the doping amount of CNTs affects the phase-transition behavior and thermal conductivity of paraffin in CPCM. Compared with pure paraffin, doping 3 wt% CNTs can increase the thermal conductivity of CPCM by 30.3%, and reduce the latent heat of melting and solidification of CPCM by 8.9% and 9.3%, respectively. KARAIPEKLI et al. [22] prepared CPCM by directly mixing uncatalyzed CNTs with porous graphite as the matrix and paraffin as the phase-change material. The thermal conductivity of CPCM increased by 113%. The experimental results indicate that mixing CNTs with porous phase-change composite materials after catalytic treatment can greatly improve the thermal conductivity of phase-change composite materials. Lin et al. [23] prepared CNTs@MXene aerogels to enhance the thermal conductivity of phase-change materials and used the composites in a battery thermal-management system, which effectively improved the uniform distribution of the battery temperature, thereby reducing the aging rate of the battery. Du et al. [24] established a molecular dynamics simulation method to predict the structure, diffusion, and thermal properties of composite phase-change materials based on CNTs. The study found that the dense phase between the CNT’s wall and PCM has been proven to be the main factor offsetting the phase-transition enthalpy of the composite system. The formation of efficient heat conduction pathways explains the underlying mechanism of the significant improvement in carbon-nanotube-based NPCM, which can be achieved under the premise of strong bonding effects of the material and reduced contact thermal resistance between CNTs in macroscopic PCM modules. Currently, research on carbon nanotube composite phase-change materials has made certain progress both domestically and internationally. By combining CNTs with PCMs, the shortcomings of the low heat-transfer efficiency of paraffin as a medium to low temperature PCMs can be alleviated to some extent.

Both the doping concentration and diameter of CNTs can affect the thermal conductivity of phase-change materials. Additionally, the number of CNT layers (single-layer vs. multi-layer) plays a critical role, as multi-layer CNTs often exhibit higher interfacial thermal resistance due to interlayer phonon scattering, while single-layer CNTs may offer more efficient heat transfer pathways [25,26]. However, current research has only discussed these factors from a single perspective, with limited studies on the comprehensive optimization of these two variables. Therefore, it is necessary to explore the optimal type of CNTs to achieve the best performance in the phase-change system. 1-Octadecanol (OD), an organic solid–liquid phase-change material, exhibits a high latent heat (240 J/g) and demonstrates stable properties under medium-to-low temperature conditions. It is non-corrosive to encapsulation materials and shows good compatibility with various encapsulants [27]. Additionally, OD can form binary, ternary, or quaternary eutectic mixtures with most monohydric alcohols, covering nearly the entire temperature range from room temperature to 100 °C. However, OD also has the disadvantage of low thermal conductivity common to organic PCMs. The low thermal conductivity adversely affects heat transfer in thermal storage systems, consequently impairing both heat charging and discharging efficiency. To address these research gaps, this study systematically investigates the influence of multi-scale CNTs on the thermophysical properties of OD phase-change heat storage materials, aiming to improve heat conduction capacity of OD. The study systematically investigated the variation patterns of microscopic morphology, phase-change temperature, phase-change enthalpies, thermal conductivity, specific heat capacity, and thermal diffusivity in CNTs/OD PCMs with different CNTs diameters (<8 nm, 10–20 nm, 20–30 nm) and concentrations (1 wt%, 3 wt%, 5 wt%, 7 wt%, 9 wt%). The optimizing effects of CNT diameter and loading content on the thermal properties of the phase-change material were analyzed. By enhancing the heat transfer during the melting of phase-change materials and further optimizing the problem-solving process, it is conducive to promoting the progress of modern renewable energy and building a resource-conserving and environment-friendly society.

## 2. Results and Discussion

### 2.1. Microstructure

**Figure 1:**
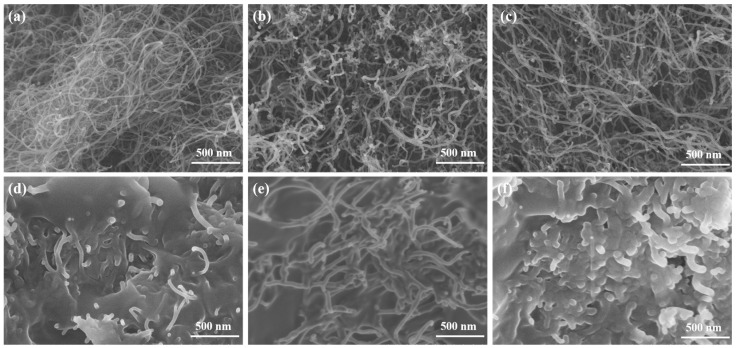
SEM images of (**a**) <8 nm CNTs, (**b**) 10–20 nm CNTs, (**c**) 20–30 nm CNTs, (**d**) OD/CNTs-A, (**e**) OD/CNTs-B and (**f**) OD/CNTs-C.

**Figure 2:**
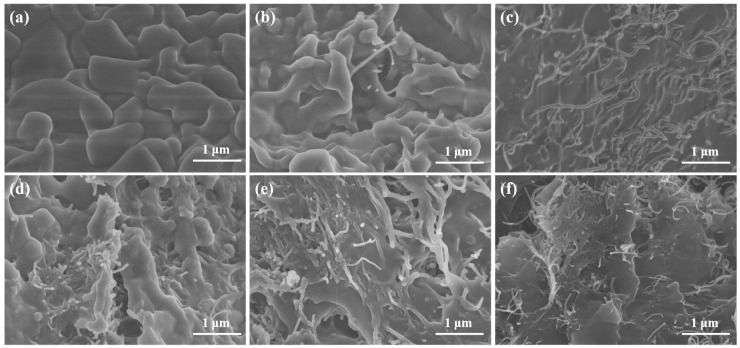
SEM images of (**a**) OD, (**b**) OD/CNTs-1, (**c**) OD/CNTs-3, (**d**) OD/CNTs-5, (**e**) OD/CNTs-7 and (**f**) OD/CNTs-9.

Figure 1a–c present SEM images of CNTs with diameters of <8 nm, 10–20 nm, and 20–30 nm, respectively. CNTs exhibit a one-dimensional tubular structure with curved morphology and uniform diameter distribution. The highly tortuous configuration of CNTs makes accurate length determination challenging. The measured fundamental parameters show good consistency with the manufacturer’s specifications. Figure 1d–f display the SEM images of OD/CNTs-A, OD/CNTs-B, and OD/CNTs-C CPCMs, respectively. Notably, the majority of CNTs show their tips and surfaces coated with OD. Morphological observations reveal that CNT agglomeration occurs, whereas individual tubular characteristics persist but lead to contact areas with limited effectiveness between adjacent tubes [28]. This inter-tube contact is further reduced due to the OD coating covering both the nanotube surfaces and tips. In the OD/CNTs-B CPCMs, although the mass fraction of CNTs is minimal, their nano-scale dimensions result in a substantial quantity of individual CNTs per unit mass. The exceptional aspect ratio of CNT units facilitates the formation of an efficient thermal conduction network within the OD matrix, demonstrating significant enhancement in thermal conductivity. Figure 2 presents the SEM images of pure OD and OD/CNTs CPCMs with varying CNT contents. As shown in Figure 2a, pure OD exhibits a bulk morphology without visible impurities. At 1 wt% CNT loading, the CNTs are uniformly dispersed in the OD matrix, though their limited quantity results in discontinuous thermal conduction networks with localized agglomeration, which may compromise the enhancement of thermal conductivity. Notably, OD/CNTs-3 demonstrates optimal dispersion characteristics among all samples. With an increase in CNT content, branched and network-like distributions emerge within the OD matrix, indicating that the dispersant effectively mitigates CNT aggregation.

### 2.2. Component

**Figure 3:**
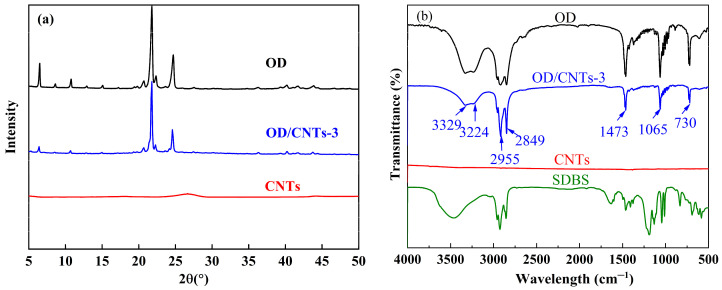
(**a**) XRD and (**b**) FTIR spectra of OD, CNTs, and OD/CNTs-3 CPCMs.

Figure 3a shows the XRD patterns of OD, CNTs, and OD/CNTs-3. The diffraction pattern reveals characteristic peaks of OD at 2θ = 6.4°, 20.6°, 21.7°, and 24.8°, which are consistent with those reported in the literature for pure OD [29]. CNTs exhibit weak diffraction peaks at 2θ = 26.5°, which is a characteristic peak of graphite caused by the (002) surface of graphite. The XRD pattern of the OD/CNTs-3 CPCMs exhibits all characteristic peaks corresponding to both OD and CNTs. This confirms that the CPCMs maintain the crystalline structure of OD while showing no additional diffraction peaks, demonstrating that the preparation process only involves physical combination without chemical reactions. The chemical structures of OD, CNTs, SDBS, and OD/CNTs-3 were investigated by FTIR spectroscopy, as shown in Figure 3b. The FTIR spectrum of OD exhibits distinct absorption peaks at 2955 cm^−1^ and 2849 cm^−1^, corresponding to the stretching vibrations of R-CH_3_ and R_2_-CH_2_ groups, respectively. A broad absorption band observed between 3329 cm^−1^ and 3224 cm^−1^ is attributed to the O-H stretching vibration. The peaks at 730 cm^−1^ and 1473 cm^−1^ are associated with in-plane and out-of-plane deformations of the hydroxyl (O-H) group, while the peak at 1065 cm^−1^ corresponds to C-O stretching vibration [30]. After compositing with CNTs, the chemical structure of OD remains unchanged, indicating excellent compatibility between OD and CNTs.

### 2.3. Thermal Storage Properties

**Figure 4:**
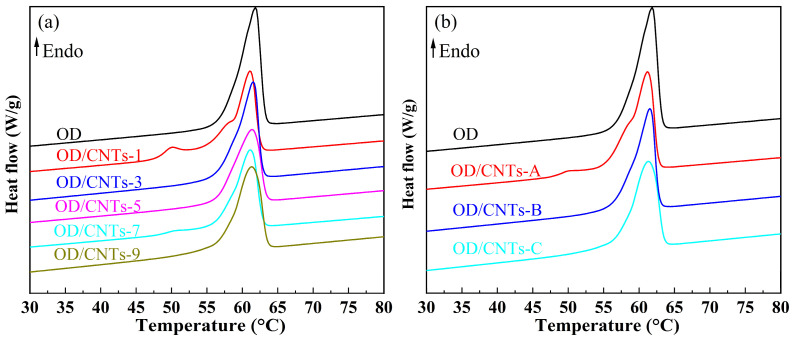
DSC melting curves of OD and OD/CNTs composite PCMs (**a**) variation with CNT content, (**b**) variation with CNT diameter.

**Figure 5:**
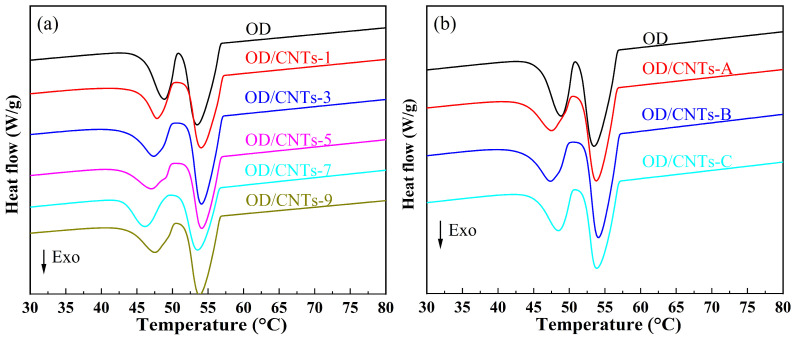
DSC solidification curves of OD and OD/CNTs CPCMs (**a**) variation with CNT content, (**b**) variation with CNT diameter.

The physical property parameters are important reference data for evaluating the thermal performance of thermal storage CPCMs. Figure 4 and Figure 5 show the DSC curves of pure OD and OD/CNTs CPCMs, which were not significantly changed by doping with CNTs. Table 1 and Table 2 present the DSC test data of pure OD and OD/CNTs CPCMs. During the melting process of OD/CNTs CPCMs, the melting temperatures of OD/CNTs-1, OD/CNTs-3, OD/CNTs-5, OD/CNTs-7, and OD/CNTs-9 were measured at 57.1 °C, 56.9 °C, 57.3 °C, 57.0 °C, and 56.8 °C, respectively. Compared with pure OD, all CNT-incorporated composites exhibited a slight reduction in melting temperature. This phenomenon can be primarily attributed to the enhanced thermal conductivity induced by the carbon-based filler. As thermal conductivity is a critical parameter in heat transfer, it plays a significant role in solid–liquid phase transitions. For the solidification process of OD/CNTs composite phase-change materials, the solidification temperatures of OD/CNTs-1, OD/CNTs-3, OD/CNTs-5, OD/CNTs-7, and OD/CNTs-9 composite phase-change materials were 56.9 °C, 57.2 °C, 57.0 °C, 57.2 °C, and 57.1 °C, respectively. The solidification temperatures of all OD/CNTs CPCMs are higher than those of pure OD. The main function of CNTs is to enhance thermal conductivity. They do not absorb or release heat during DSC testing. The latent heat of melting and solidification of OD/CNTs CPCMs are mainly contributed by OD. The melting enthalpies (ΔH_m_) of the OD/CNTs-1, OD/CNTs-3, OD/CNTs-5, OD/CNTs-7, and OD/CNTs-9 CPCMs were 238.8 J/g, 233.0 J/g, 227.1 J/g, 224.9 J/g, and 218.5 J/g, respectively, while their solidification enthalpies (ΔHs) were 207.0 J/g, 202.9 J/g, 198.6 J/g, 193.2 J/g, and 190.0 J/g, respectively. The measured values showed minimal deviation from the theoretical melting and solidification enthalpies. From Table 2, it can be seen that as the diameter of the CNT increases, both the phase-transition temperature and latent heat decrease. The phase-transition temperature decreases by about 1 °C, and the latent heat decreases by 2.3–4.6 J/g. The primary reason is the intermolecular adhesion. The addition of CNTs reduces the cohesive forces between OD molecules, leading to a less ordered molecular arrangement. Moreover, as the diameter of the CNT increases, their specific surface area decreases, making it harder for OD molecules to adsorb onto the CNT surfaces. This promotes a transition from an ordered to a disordered state in OD molecules, thereby lowering the phase-transition temperature. Additionally, larger-diameter CNTs exhibit a smaller specific surface area, which further weakens the adsorption capacity and binding force for OD molecules. As a result, during melting, the molecular alignment of OD undergoes more significant disruption, leading to a reduction in the phase-change enthalpy. Overall, the addition of CNTs does not reduce the thermal properties of OD matrix much, and the latent heat of phase transition still has a high value, which is more than 190 J/g. The suitable phase-transition temperature and high latent heat of phase transition are suitable for medium and low temperature solar thermal utilization.

### 2.4. Heat Transfer Performance Analysis

**Figure 6:**
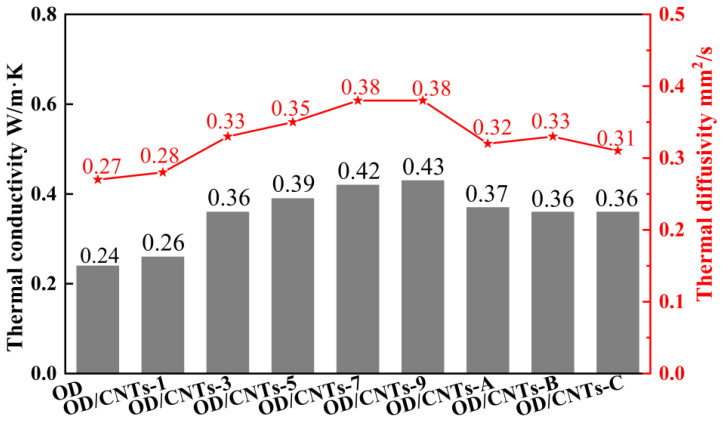
Thermal diffusivity and thermal conductivity of OD and OD/CNTs CPCMs.

**Figure 7:**
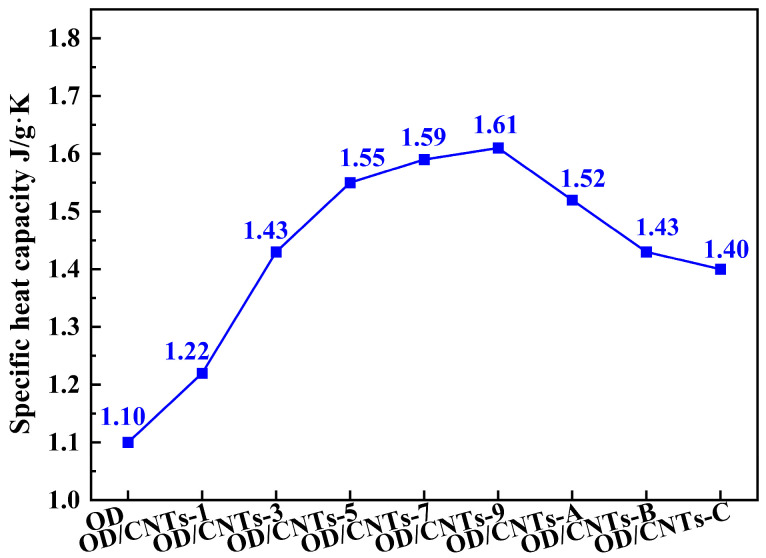
Specific heat capacity of OD and OD/CNTs CPCMs.

Figure 6 presents the thermal diffusivity and thermal conductivity test data for pure OD and OD/CNTs CPCMs. As can be observed, both the thermal diffusivity and thermal conductivity of the OD/CNTs increase with higher loading amounts of CNTs. The pure OD exhibited thermal diffusivity and thermal conductivity values of 0.27 mm^2^/s and 0.24 W/m·K, respectively. In comparison, the OD/CNTs CPCMs demonstrated significantly enhanced thermal properties. The thermal diffusion coefficients of the OD/CNTs-1, OD/CNTs-3, OD/CNTs-5, OD/CNTs-7, and OD/CNTs-9 CPCMs are 0.28 mm^2^/s, 0.33 mm^2^/s, 0.35 mm^2^/s, 0.38 mm^2^/s, and 0.38 mm^2^/s respectively. The thermal conductivities were 0.26 W/m·K, 0.36 W/m·K, 0.39 W/m·K, 0.42 W/m·K, and 0.43 W/m·K, respectively. All composite materials showed superior thermal performance compared to pure OD, with both thermal diffusivity and conductivity increasing progressively with higher CNT loading. The thermal conductivities of the three diameters of CNTs at a mass fraction of 3 wt% range from 0.36 to 0.37 W/m·K, which is 1.5 times the thermal conductivity of pure OD. The thermal conductivity enhancement of the composite exhibited a nonlinear relationship with CNT concentration, while higher CNT loading (9 wt%) improved heat transfer by 180%. The OD/CNTs-3 CPCMs exhibit thermal diffusivity and thermal conductivity values that are 1.2 times and 1.5 times higher than those of pure OD, respectively. This result clearly demonstrates that CNTs can effectively enhance the thermal conduction capability of pure OD. The OD/CNTs-9 CPCMs demonstrate thermal diffusivity and thermal conductivity values that are 1.4 times and 1.8 times those of pure OD, respectively. This phenomenon can be explained by the microstructure of the OD/CNTs-9 composite: 1. Carbon nanotube morphology effects. The CNTs in this composite feature relatively long lengths and high curvature. Their nano-scale effects promote aggregation, forming clustered OD/CNTs domains. Even when partial connections exist between nanotubes within these clusters, the interconnectedness between different clusters remains limited. 2. OD coating limitations. Although the CNTs are interconnected through OD coating, the highly thermally conductive CNT walls and tips become separated by OD layers. This reduces the effective contact area between CNTs and increases interfacial thermal resistance. 3. Heat transfer pathway challenges. The severely curved CNTs prolong heat transfer pathways, thereby impeding efficient thermal transport. These observations align well with the SEM images of the OD/CNTs-9 composite, confirming the proposed microstructure–property relationship. Figure 7 presents the specific heat capacity data of pure OD and OD/CNTs CPCMs. The specific heat capacity of OD/CNTs CPCMs initially increases with carbon nanotube loading, eventually stabilizing at 1.6 J/(g·K). This thermal enhancement can be attributed to the unique microstructure. CNTs with high specific surface areas form continuous, three-dimensional thermally conductive pathways throughout the composite matrix, and the interconnected CNT framework provides multidirectional heat transfer channels, which creates spatial thermal networks with large length–width–height ratios. This structural configuration offers an effective approach for improving thermal conductivity in shape-stabilized phase-change energy storage materials. OD/CNTs-A, OD/CNTs-B, and OD/CNTs-C have identical CNT content, and the decrease in specific heat capacity is primarily attributed to the increased diameter of CNTs. This dimensional change reduces their specific surface area, consequently decreasing the available heat transfer area and leading to diminished specific heat capacity [31,32]. The thermal conductivity value is comparable to similar CNT-PCM composites [33,34,35]. However, our system outperforms in energy density and cycling stability, making it preferable for applications prioritizing capacity over rapid heat transfer.

### 2.5. Reliability and Reversible Stability

**Figure 8:**
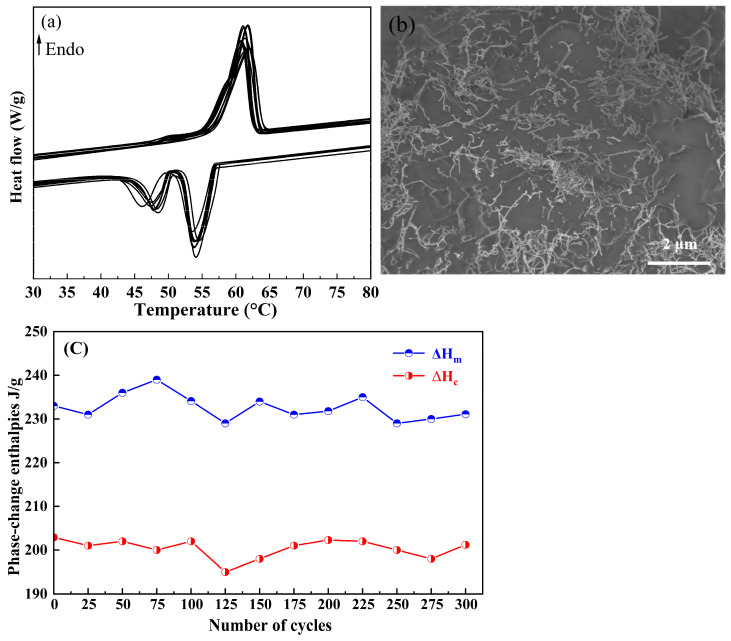
(**a**) Multicycle DSC thermograms. (**b**) SEM images and (**c**) phase-change enthalpies of OD/CNTs-3 as a function of cycle number obtained from multicycle DSC scans.

Excellent reliability and stability are critical issues that phase-change materials must address for long-term use, requiring their thermal properties and chemical structure to remain stable. In order to investigate the cycling stability of OD/CNTs CPCMs, 300 cycles of 30–90–30 °C thermal cycling tests were performed on the OD/CNTs CPCMs. The phase-transition temperature and latent heat of phase transition after the cycle were tested and recorded at intervals of 25 times, respectively. The results are shown in Figure 8. As can be seen from Figure 8a, with increasing thermal cycles, all DSC curves maintained consistent peak shapes and numbers, with only slight variations in peak area. It is worth noting that there is a minor reduction in thermal enthalpies at cycle 125. Localized delamination occurred at CNT-PCM interfaces due to accumulated thermal stress [36]. From Figure 8b, SEM characterization after 300 thermal cycles confirms that the CNTs remain uniformly dispersed within the OD/CNTs CPCMs without significant agglomeration, demonstrating excellent structural stability. Table 3 shows that after 300 thermal cycles, the melting temperature of OD/CNTs-3 remained at 56.8 °C, while its phase-change latent heat was 231.0 J/g, representing a mere 0.8% reduction. Moreover, the OD/CNTs CPCMs demonstrated excellent stability in thermal conductivity before and after the cycling tests.

### 2.6. Heat Storage and Release Performance

**Figure 9:**
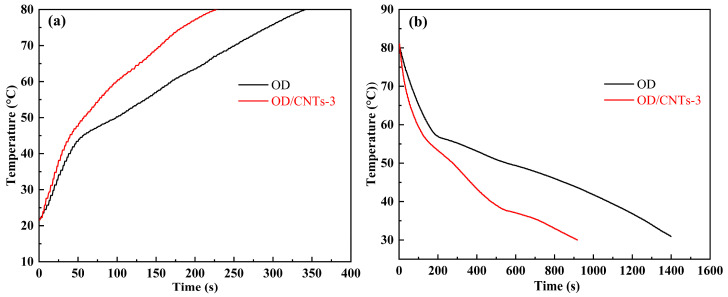
(**a**) Heat storage and (**b**) release curves of OD and OD/CNTs-3 CPCMs.

The main purpose of the heating and cooling experiment is to measure the heating/cooling time and efficiency of pure OD and CPCMs, serving as an important means to characterize thermal energy storage efficiency. The heating and cooling curves provide the most intuitive representation of the PCM’s thermal energy storage capacity, making this performance test highly significant. Figure 9a,b present the heating and cooling curves of OD and OD/CNTs-3, respectively. The capacity and efficiency for effective heat storage and release are crucial for thermal energy storage systems. As can be seen from Figure 9a, from the onset of heating, the temperature rise curve of the OD/CNTs-3 CPCMs runs slightly higher than that of OD. The heating rate of OD/CNTs-3 is also faster than that of OD. When the temperature increases to 70 s, the temperature of OD/CNTs-3 significantly surpasses that of OD. The OD/CNTs-3 required 82 s to reach its melting point (56.9 °C), whereas OD needed 136 s to heat from room temperature to the same temperature. With the addition of 3 wt% CNTs, the heating time of the OD/CNTs-3 was reduced by 39.7%. As shown in Figure 9b, the cooling rate of the OD/CNTs-3 consistently exceeds that of OD from the beginning of the cooling process. The OD/CNTs-3 required only 720 s to cool to 35 °C, compared to 1266 s for OD to reach the same temperature from room temperature. With the incorporation of 3 wt% CNTs, the cooling time of the OD/CNTs-3 was reduced by 43.1%. CNTs may enhance heat dissipation in composites by providing high-axial thermal conductivity pathways, which is critical for rapid heating and cooling. In addition, poor dispersion of CNTs in the matrix can create thermal barriers in heating scenarios but may matter less in cooling [37,38]. In general, incorporating CNTs improves the heating and cooling rates of the CPCMs.

Compared with OD, the OD/CNTs-3 showed only a modest reduction in heating time, which is consistent with its thermal diffusivity and conductivity test data. While CNTs possess intrinsically high thermal conductivity, the OD/CNTs CPCMs did not exhibit orders-of-magnitude enhancement in either thermal conductivity or specific heat capacity. Consequently, the improvements in heat charge/discharge rates, though measurable, remain modest. After incorporating CNTs, the high degree of curvature and nano-scale effects lead to significant entanglement within the OD/CNTs-3 CPCMs. In this system, CNTs tend to aggregate into clusters where their tips and sidewalls become coated with OD molecules. This coating prevents direct contact between adjacent CNTs, thereby reducing their effective contact area. Furthermore, the OD matrix between CNTs further impedes heat transfer. Additionally, the likelihood of interconnected networks forming between these CNT-rich clusters is relatively low. In summary, the presence of CNTs provides only minimal enhancement to the thermal conductivity of OD. Since the liquid-state thermal conductivity of the OD/CNTs CPCMs could not be experimentally measured, we hypothesize that the observed phenomenon may arise from the following mechanism: During the heating process, the increased fluidity of the molten material allows CNTs greater freedom of movement. This enhanced mobility enables CNTs reorganization and improved inter-tube connections, thereby increasing the effective contact area and rectifying previously inefficient conductive pathways. Consequently, the time reduction observed during the cooling process is more pronounced than that during the heating process.

## 3. Materials and Methods

### 3.1. Materials

**Figure 10:**
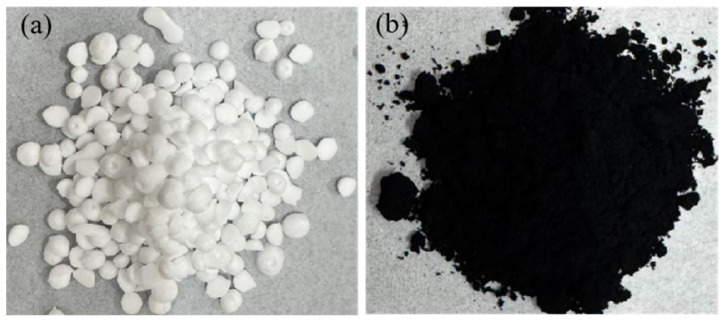
Pictures of (**a**) OD and (**b**) CNTs-B.

1-Octadecanol (OD, C_18_H_38_O) was produced by Sinopharm Chemical Reagent Co., Ltd. (Beijing, China).; multi-wall carbon nanotubes (CNTs) were purchased from Beijing Boyu Gaoke New Material Technology Co., Ltd. (Beijing, China). (>95 wt%, diameter < 8 nm, 10–20 nm, 20–30 nm; length 10–30 μm); and sodium dodecyl benzene sulfonate (SDBS, analytical grade) employed as a dispersant in this study, was obtained from Sinopharm Chemical Reagent Co., Ltd. (Beijing, China). The pictures of OD and CNTs-B were showed in Figure 10. All materials were used as received without further purification.

### 3.2. Preparation of OD/CNTs CPCMs

A total of 10 g of OD was melted at 80 °C in a beaker. Then, 1 wt% CNTs and equivalent SDBS were slowly added to prevent agglomeration. The sulfonate group (-SO_3_^−^) of SDBS adsorbs onto CNT surfaces via π–π stacking, while the dodecyl tail extends into the solvent, creating electrostatic and steric repulsion. SDBS enhances the homogeneous distribution of CNTs through electrostatic and steric stabilization, thereby establishing interconnected networks for efficient heat transfer. The mixture was dispersed uniformly using magnetic stirring for 20 min. Subsequently, CNTs were dispersed via pulsed ultrasonication (600 W, 40 kHz, 1 h with 10 min on/5 min off cycles) to ensure uniform matrix distribution. Finally, the composite was allowed to solidify through natural cooling. The above procedure was repeated to prepare OD-based CPCMs containing 1 wt%, 3 wt%, 5 wt%, 7 wt%, and 9 wt% CNTs, which were designated as OD/CNTs-1, OD/CNTs-3, OD/CNTs-5, OD/CNTs-7, and OD/CNTs-9 CPCMs, respectively. OD/CNTs CPCM with different tube diameters and an addition amount of 3 wt% were prepared by the same method. The samples with CNT diameters of <8 nm, 10–20 nm, and 20–30 nm were named OD/CNTs-A, OD/CNTs-B, and OD/CNTs-C CPCMs, respectively. The detailed mass of OD/CNTs CPCM is shown in Table 4. It is worth noting that OD/CNTs–B and OD/CNTs-3 refer to the same sample (3 wt% CNT loading, 10–20 nm diameter) and thus share identical experimental data.

### 3.3. Characterization

The surface microstructure, organization, and morphology of the materials were characterized using a Japanese Electronics field-emission scanning electron microscope (SEM, JSM-7500F, JEOL, Tokyo, Japan). Phase identification and quantitative analysis were performed by X-Ray diffraction (Empyrean, Bruker, Bremen, Germany) with a 2θ goniometer range of 5°–60°. Molecular structure analysis was conducted via Fourier-transform infrared spectroscopy (Nicolet 6700, Madison, WI, USA) to obtain the infrared spectra of the samples. Test wavelength range was at 5000–350 cm^−1^, resolution 4 cm^−1^. The DSC (NETZSCH, Selb, Germany) measurements were conducted from room temperature to 100 °C at a heating rate of 10 °C/min under a nitrogen atmosphere with a flow rate of 10 mL/min. The temperature accuracy is ±0.2 °C. The thermal conductivity, thermal diffusivity, and specific heat capacity of the CPCMs were determined using a Hot Disk thermal constants analyzer (Hot Disk AB, Gothenburg, Sweden) with 7577 sensor type and 50 mW power. The uncertainty analysis of instrumentation is ±2%. To ensure data reliability, each sample was measured three times and the average value was calculated. Heat storage and release curves were measured through the following steps. An adequate amount of the test material is loaded into a test tube, and a standard thermocouple mounted on an intelligent temperature controller is inserted, ensuring the material fully covers the thermocouple. The test tube is then secured in a thermostatic heating magnetic stirrer with a built-in heating bath, making sure the sample height remains below the liquid surface. The liquid temperature is controlled to uniformly heat the sample, while temperature data is collected via the intelligent temperature controller. Data acquisition is stopped once the sample completes the phase transition and stabilizes in temperature, after which the data is exported to plot the heating curve. Similarly, the test material is heated to the same temperature above its melting point and then allowed to cool naturally in air to room temperature. Temperature data is collected via the intelligent temperature controller until the sample cools to ambient conditions, after which the data is exported to plot the cooling curve.

## 4. Conclusions

This study selected CNTs as additives to prepare OD/CNTs composite phase-change materials. The research systematically investigated the effects of incorporating CNTs with different diameters and additions on the phase composition, phase-transition temperature, phase-change enthalpy, thermal diffusivity, thermal conductivity, thermal stability, and heating/cooling duration of OD-based PCMs. Furthermore, the microstructural characteristics of the OD/CNTs CPCMs were analyzed to elucidate the underlying mechanisms responsible for the observed modifications in thermophysical properties. Ultimately, this work establishes fundamental structure–property relationships between composition, microstructure, and performance in OD-based PCMs.

(1) The XRD pattern of the OD/CNTs simply represents a superposition of the individual diffraction patterns of CNTs and OD, demonstrating that only physical mixing occurs between the components without any chemical reaction.
(2) The incorporation of CNTs showed negligible effects on the melting and solidification temperatures of OD. Notably, the OD/CNTs composites maintained high latent heat values for both phase transitions. Specifically, the OD/CNTs-3 composite exhibited melting and solidification temperatures of 56.9 °C and 57.2 °C, respectively, with corresponding latent heats of 233.0 J/g and 202.9 J/g. The OD/CNTs-3 CPCMs demonstrated significant thermal enhancement, exhibiting thermal diffusivity and conductivity values 1.2 times and 1.5 times higher than pure OD, respectively.
(3) With increasing CNT loading, the OD/CNTs CPCMs exhibited a marginal reduction in heat storage capacity, while both thermal conductivity and thermal diffusivity demonstrated linear enhancement.
(4) The composite showed remarkable improvements in thermal cycling efficiency, with heating time reduced by 39.7% and cooling time decreased by 43.1% compared to the OD. The CNTs formed an efficient thermal conduction network within the OD, significantly reducing both the heating and cooling durations of the PCMs.

This modification strategy using CNTs proves to be a viable approach, demonstrating considerable potential for advancing thermal energy storage applications.

(1) For electronic thermal management, it serves as chip-level phase-change cooling modules capable of absorbing transient high heat flux.
(2) In solar energy systems, it significantly enhances intermittent energy utilization efficiency when coupled with solar collectors for low-temperature thermal storage.

To advance the practical application of this composite, two critical areas warrant further investigation: (1) scale-up production methods, focusing on optimizing batch consistency and homogeneity in large-volume manufacturing; (2) long-term corrosion/oxidation studies, particularly under cyclic thermal stress and varying humidity conditions to evaluate decade-scale durability.

## Figures and Tables

**Table 1:** DSC data of OD and OD/CNTs CPCMs with different CNT content.

Samples	Melting		Solidifying	
	T_m_ (°C)	ΔH_m_ (J/g)	T_s_ (°C)	ΔH_s_ (J/g)
OD	57.7	242.2	56.7	210.1
OD/CNTs-1	57.1	238.8	56.9	207.0
OD/CNTs-3	56.9	233.0	57.2	202.9
OD/CNTs-5	57.3	227.1	57.0	198.6
OD/CNTs-7	57.0	224.9	57.2	193.2
OD/CNTs-9	56.8	218.5	57.1	190.1

**Table 2:** DSC data of OD and OD/CNTs CPCMs with different CNT diameter.

Samples	Melting		Solidifying	
	T_m_ (°C)	ΔH_m_ (J/g)	T_s_ (°C)	ΔH_s_ (J/g)
OD/CNTs-A	56.6	234.6	55.3	203.1
OD/CNTs-B	56.9	233.0	57.2	202.9
OD/CNTs-C	56.0	231.5	55.5	201.6

**Table 3:** Thermal properties before and after different thermal cycles of OD/CNTs-3.

Samples	Melting		Solidifying		K (W/m·K)
	T_m_ (°C)	ΔH_m_ (J/g)	T_s_ (°C)	ΔH_s_ (J/g)	
OD/CNTs-3(0)	56.9 ± 0.3	233.0	57.2 ± 0.1	202.9	0.36
OD/CNTs-3(100)	56.6 ± 0.2	234.1	57.1 ± 0.2	202.0	0.36
OD/CNTs-3(200)	57.1 ± 0.1	231.8	56.8 ± 0.3	202.3	0.35
OD/CNTs-3(300)	56.8 ± 0.2	231.1	56.6 ± 0.2	201.2	0.35

**Table 4:** The mass of OD/CNTs CPCM.

Samples	OD/g	Diameter of CNTs/nm	CNTs/g	SDBS/g
OD/CNTs-1	10	10–20	0.1	0.1
OD/CNTs-3	10	10–20	0.3	0.3
OD/CNTs-5	10	10–20	0.5	0.5
OD/CNTs-7	10	10–20	0.7	0.7
OD/CNTs-9	10	10–20	0.9	0.9
OD/CNTs-A	10	<8	0.3	0.3
OD/CNTs-B	10	10–20	0.3	0.3
OD/CNTs-C	10	20–30	0.3	0.3

## Data Availability

Data will be made available on request.

## Abbreviations

The following abbreviations are used in this manuscript:

PCMs	Phase-change materials
CNTs	Carbon nanotubes
OD	1-octadecanol
CPCMs	Composite phase-change materials
XRD	X-Ray diffraction
FTIR	Fourier-transform infrared spectroscopy
SEM	Scanning electron microscopy
DSC	Differential Scanning Calorimeter
SDBS	Sodium dodecyl benzene sulfonate
ΔH_m_	Melting enthalpies
ΔH_s_	Solidification enthalpies
